# Meticulous Surgical Excision of a Localized Giant Cell Tumor of the Tendon Sheath

**Published:** 2013-03-13

**Authors:** Deepa Cherla, Edward Hahn, Ramazi Datiashvilli

**Affiliations:** New Jersey Medical School, University of Medicine and Dentistry of New Jersey, Newark

**Figure:**
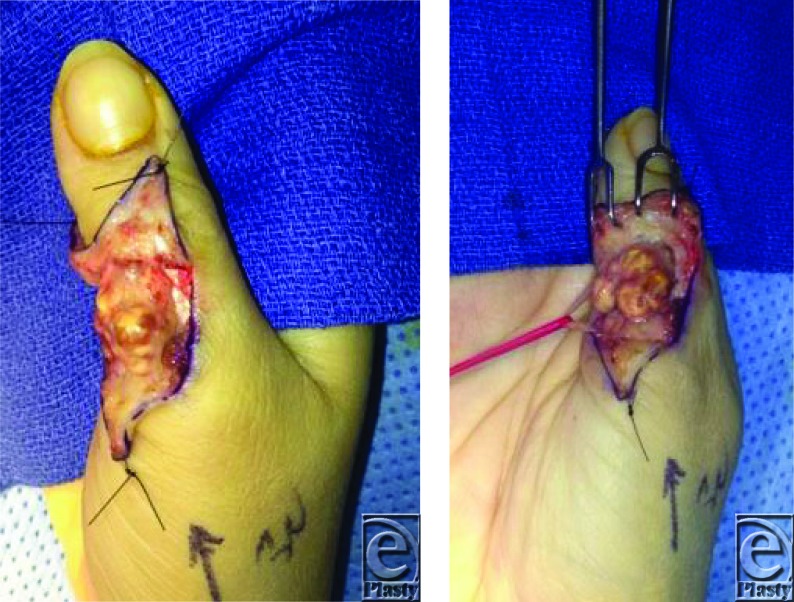


## DESCRIPTION

A 32-year-old right hand dominant female presented with a 2-year history of a slowly growing firm, nodular, and nontender right thumb mass. Sensation and strength in the involved digit were intact. Range of motion was mildly restricted. Surgical dissection of the lesion is shown in Figures.

## QUESTIONS

1. **What is the differential diagnosis?**
2. **What are the clinical and morphological characteristics of this condition?**
3. **What are the treatment options?**
4. **What are current recommendations for preventing recurrences?**

## DISCUSSION

The differential diagnosis of this patient's hand lesion included giant cell tumor of the tendon sheath (GCTTS), ganglion, synovial cyst, fibrous histiocytoma, and nodular fasciitis.^1^ Malignant neoplasms, such as synovial sarcoma, malignant fibrous histiocytoma, and clear cell sarcoma, were less probable diagnoses due to the lack of calcifications, infiltration of bone, or pathologic fractures. Radiographs of the right hand and wrist showed a well-defined pressure erosion consistent with a long-standing soft tissue neoplasm wrapping around the first proximal phalanx anteriorly, across the radial cortex, and posteriorly. Magnetic resonance imaging of the right upper extremity revealed a well-defined nonencapsulated soft tissue neoplasm surrounding the body of the first proximal phalanx of the right thumb that was 1.8 cm × 1.6 cm × 1.8 cm. This lesion was located between the posterior cortex of the proximal phalanx and the flexor pollicis longus, with the radial aspect of the lesion overlying the extensor expansion of the extensor pollicis longus, with no infiltration of digital neurovascular bundles, tendons, joints, or the medullary region of the proximal phalanx. This patient's clinical and radiologic findings were most consistent with GCTTS.

First described by Chassaignac in 1852,^2^ GCTTS, otherwise known as pigmented villonodular proliferative tenosynovitis, is a benign tumor that is best considered idiopathic.^3^^,^^4^ GCTTS is the second most common mass of the hand after ganglion cysts^2^ and is most frequently diagnosed in the fourth and fifth decades of life, with women affected more commonly than men.^5^ Grossly, GCTTS is a multilobular and well-circumscribed tumor that often has a bosselated or clefted outer surface. Coloration varies from gray to golden to yellow-orange to tan to red-brown, depending on hemosiderin, collagen, and histiocyte content. Because of the slow-growing nature of localized tumors, patients usually present 6 months to 2.5 years after the initial onset of symptoms.^2^^,^^6^ Morphologically, 2 variants of GCTTS exist—a localized nodular type (type I) more common in the hand and a diffuse type (type II) more common in larger joints.^5^^,^^7^^-^8 The diagnosis of GCTTS is largely made by clinical examination and various imaging modalities, but definitive diagnosis is deduced by tissue biopsy and may be made through the examination of intraoperative findings.^2^ The most definitive imaging study for diagnosis is contrast-enhanced magnetic resonance imaging, which can identify the extent of the soft tissue mass, satellite lesions, or bone marrow changes and usually shows hypointense lesions on T1- and T2-weighted images.^2^^,^^3^

Treatment of localized GCTTS consists of complete open or arthroscopic resection of the tumor as well as all residual satellite nodules or intraosseous lesions. The open approach with wide surgical exposure and meticulous dissection with an operating microscope or magnifying loupes is preferred to prevent contamination and reduce recurrence rates.^8^^,^^9^ Care must be taken to preserve surrounding structures not invaded by the GCTTS, including tendons, digital arteries, and nerves.^2^ Radiotherapy may decrease recurrence rates when used as adjuvant therapy but is not frequently advocated for joints of the hand.^2^^,^^8^

Following surgical excision, GCTTS has a reported recurrence rate of 4% to 44%. Recurrence is most strongly associated with incomplete excision and is more variably associated with the presence of satellite, diffuse-type, or multicentric lesions; intraosseous invasion; and direct extension of the tumor to tendons, the joint capsule, or the distal or thumb interphalangeal joints.^2^^,^^3^^,^^7^^,^^9^ The lowest recurrence rates are associated with type 1 tumors and complete excision of the mass under magnification.^7^ Marginal excision of the tumor should be repeated in cases of recurrence.^2^

**Figure 1:**
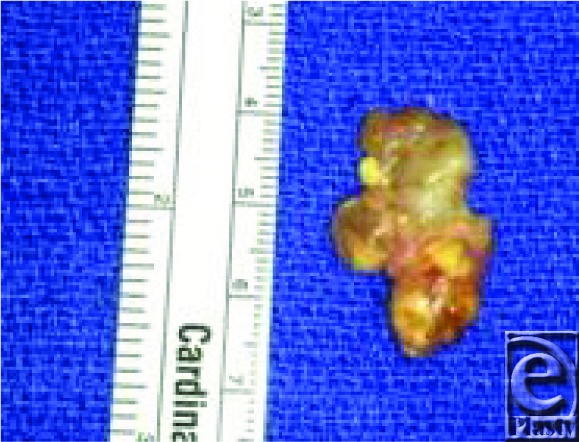
The excised giant cell tumor.

This patient's GCTTS was more consistent with a type I lesion. During gentle dissection of the patient's GCTTS lesion, the golden to yellow tumor (Fig 1) was found to be closely associated with the radial digital nerve with unclearly delineated extension into the surrounding soft tissues. The digital nerve was successfully dissected from the tumor using loop magnification with no complications or nerve damage. The tumor and adjacent soft tissues were carefully excised. The tumor also appeared to contact the bone of the proximal phalanx and adjacent tendon but did not appear to penetrate either structure. The patient's postoperative course was uncomplicated.
